# Report of the 2023 Mary Tyler Moore Vision Initiative Workshop

**DOI:** 10.1167/tvst.14.7.11

**Published:** 2025-07-18

**Authors:** S. Robert Levine, Dorene S. Markel, Martin G. Myers, Ryan Barunas, Jennifer K. Sun, Dolly S. Chang, Maureen G. Maguire, Stela Vujosevic, Patrice E. Fort, Mark Atkinson, Fabio Baschiera, Chris Gates, Judy M. Hunt, George L. King, Matthias Kretzler, Phillip Lai, Drew Lewis, Ulrich F. O. Luhmann, Stephen McLeod, Carol Oxenreiter, Shelby Unsworth, Wiley Chambers, Lloyd Paul Aiello, Jason Ehrlich, Chris L. German, Ramin Tadayoni, Risa M. Wolf, Paolo Silva, Eric Carlson, Brian Gott, Monica Oxenreiter, Nicole Sexton, Arup Das, Thomas W. Gardner

**Affiliations:** 1 Mary Tyler Moore Vision Initiative, Greenwich, CT, USA; 2 Mary Tyler Moore Vision Initiative and Department of Learning Health Sciences, University of Michigan, Ann Arbor, MI, USA; 3 Caswell Diabetes Institute, University of Michigan, Ann Arbor, MI, USA; 4 JDRF Northeast Ohio & Michigan Chapter, Southfield, MI, USA; 5 Joslin Diabetes Center, Harvard Medical School, Boston, MA, USA; 6 Kodiak Sciences, Palo Alto, CA, USA; 7 University of Pennsylvania, Philadelphia, PA, USA; 8 JAEB Center for Health Research, Tampa, FL, USA; 9 Eye Clinic, IRCCS MultiMedica, Milan, Italy; 10 Department of Biomedical, Surgical and Dental Sciences, University of Milan, Milan, Italy; 11 Department of Ophthalmology & Visual Sciences, Kellogg Eye Center, University of Michigan, Ann Arbor, MI, USA; 12 Department of Pathology and Diabetes Research Institute, University of Florida, Gainesville, FL, USA; 13 Bayer Pharmaceuticals, Basel, Switzerland; 14 Bioinformatics Core, University of Michigan, Ann Arbor, MI, USA; 15 Lay Advisor MTM Vision, Villanova, PA, USA; 16 Division of Nephrology, Department of Internal Medicine, University of Michigan, Ann Arbor, MI, USA; 17 Perfuse Therapeutics, San Francisco, CA, USA; 18 Estenda Solutions, Wayne, PA, USA; 19 Roche Pharmaceutical Research and Early Development, Translational Medicine Ophthalmology, Roche Innovation Center, Basel, Switzerland; 20 American Academy of Ophthalmology, San Francisco, CA, USA; 21 Lay Advisor, MTM Vision, Hilton Head, SC, USA; 22 Fast Forward Medical Innovation, University of Michigan Medical School, Ann Arbor, MI, USA; 23 Food and Drug Administration, Silver Spring, MD, USA; 24 Ollin Biosciences, Austin, TX, USA; 25 Myriad Genetics, Salt Lake City, UT, USA; 26 Université Paris Cité, Lariboisière Hospital, Paris, France; 27 Saint Louis Hospital, Paris, France; 28 Division of Endocrinology and Diabetes, Department of Pediatrics, Johns Hopkins University Hospital, Baltimore, MD, USA; 29 The Carlson Company, Los Angeles, CA, USA; 30 Entertainment Industry Foundation, Los Angeles, CA, USA; 31 Close Concerns, San Francisco, CA, USA; 32 Department of Ophthalmology, University of New Mexico Medical School, Albuequerque, NM, USA

## Abstract

The Mary Tyler Moore Vision Initiative (MTM Vision) Diabetic Retinal Disease (DRD) Clinical Endpoints Workshop was held on November 14, 2023. More than 130 clinicians, scientists, and representatives from funding and regulatory agencies, diagnostic, therapeutic, and biotech industry and patient advocates discussed the needs for new diagnostic and therapeutic approaches to preserve and restore retinal neurovascular unit integrity in people with diabetes. MTM Vision projects, notably updating the DRD staging system and severity scale, establishing a human ocular biorepository and resource, and validating useful clinical endpoints and biomarkers to accelerate development of new drugs and improve patient care were emphasized. A public-private consortium is essential to fulfill the objectives of MTM Vision for the benefit of persons with diabetes.

## Introduction

Martin Myers and Dorene Markel of the Caswell Diabetes Institute and Mary Tyler Moore Vision Initiative, respectively, opened the second Mary Tyler Moore Vision Initiative workshop. They urged people to work together like rowing teams to shift paradigms and make important impacts on patient care and outcomes.

### S. Robert Levine

As the coxswain my job is to shout, “Row, row, row.” Mary, my wife, and I were together for nearly 35 years, and she suffered greatly from type 1 diabetes and its complications, but her dream was to help find cures for diabetes, diabetes complications, and to help find cures and prevent vision loss due to diabetes.

The MTM Vision Initiative is a unique nonprofit organization whose mission is to accelerate development of new ways to preserve and restore vision in people with diabetes. We launched this program to honor Mary and realize her dream of a world without vision loss from diabetes.

Our purpose starts with people and the impacts of vision loss and our desire to ensure that people with diabetes can be free of those concerns. It is about accelerating progress by eliminating barriers, providing needed tools and resources, and linking them to collaborative research networks that share their data.

I used to talk about scientists saying, “If only I had this, then we could do that.” We're about providing the answer to the “if only” question with critical path research resources so people can choose to invest their time and dollars in solving vision loss from diabetes. Our top-line strategic goals include convening the global community of relevant experts, enabling detailed understanding of pathophysiology, progression and regression of diabetic retinal disease, fostering collaboration, data sharing, and establishing standardized approaches to data analysis. We're trying to provide the tools and the pathways for the science to accelerate.

Our phase one projects: (1) the update of the Diabetic Retinal Disease (DRD) staging system; (2) our ocular biorepository; and (3) our clinical endpoints project. We are defining new indications for therapeutics development including for earlier-stage disease, enabling identification of new therapeutic targets at the molecular and cellular level, and informing clinical care and regulatory pathways for new therapeutics.

For example, the Figure illustrates how the current staging system is equivalent to the 1960s rotary phone. It was unidimensional but effective. Today we need to create the multidimensional smartphone version of DRD staging, which includes the vascular and neural retina, the basic and cellular environment, systemic factors, visual function, and quality of life.

**Figure.**
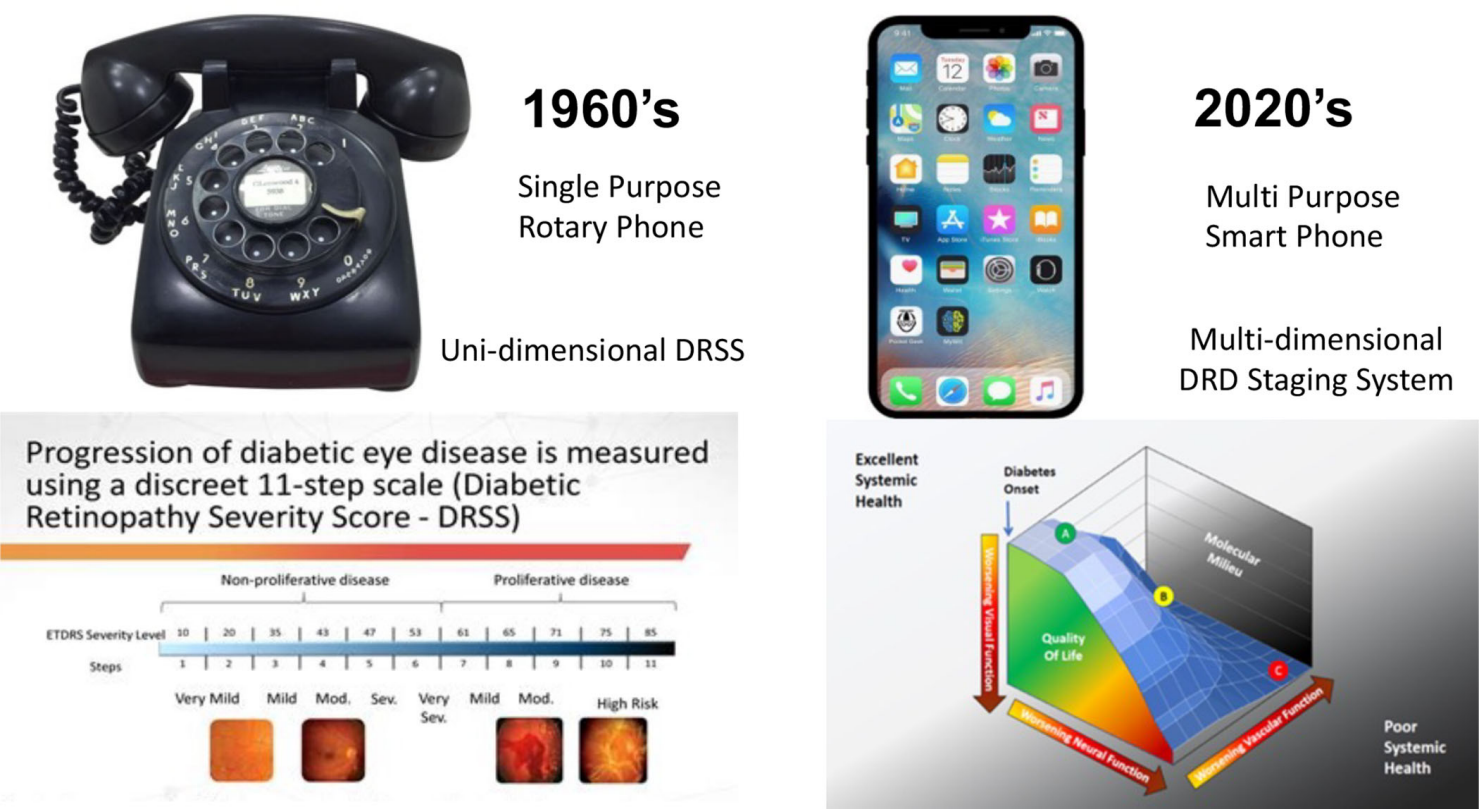
Advances in communication and diabetic retinopathy grading. The unidimensional rotary telephone was state of the art communication in the 1960s when the Airlie House classification and its derivative, the Diabetic Retinopathy Severity Score, were developed. In the 21st century smart cell phones are state-of-the-art, and a proposed three-dimensional diabetic retinal disease scoring system has been proposed in which visual function, vascular, and neural function are quantified.^1^

The task is challenged by the fact that people who do not have photographic evidence of vascular disease and those with nonproliferative diabetic retinopathy (NPDR) may have measurable functional deficits. Can we identify advanced measures of disease that can be used for clinical endpoints to evaluate new therapeutics targeted at those earlier stages of disease with the hope of preventing progression? What are the considerations? Patient burden of new tests, device availability, Food and Drug Administration (FDA) certification, and good test-retest reliability, both quantitative and objective.

To build awareness, we have a strong relationship with *Variety* magazine and are aligned with *Being Mary Tyler Moore*, the documentary I executive-produced with HBO and Lena Waithe. The Entertainment Industry Foundation and Eric Carlson are exploring a recreation Mary's *Mary Tyler Moore Show* to sell to networks and corporate sponsors to help us raise dollars for the Vision Initiative. Please visit our website at www.marytylermoore.org.

Ryan Barunas detailed his experience with diabetic retinopathy in spite of doing what he could do to control his diabetes. He received injections and scatter laser therapy in both eyes, and ultimately required vitrectomies. The experience was catastrophic for him. He still deals with constant floaters, a narrow field of vision and night glare in both eyes. He has learned to deal with the situation, but it's not a desired outcome, and he hopes this initiative will help lead to better outcomes for patients. He emphasized that patients rely on members of the audience to improve the prevention, diagnosis, and treatment of diabetes-related eye disease.

### Clinical Studies on Useful Endpoints and Biomarkers for DRD

#### Update of the DRD Staging System

Jennifer Sun explained how the case for a revised DRD grading scale was developed.^1^^,^^2^ Best-corrected visual acuity and the Diabetic Retinopathy Severity Scale do not inform about the neurovascular aspects of DRD pathophysiology or address regression of DRD. Optical coherence tomography (OCT) does not provide information about visual function.

Six working groups undertook narrative reviews to understand the variables that might rise to the top to create a multidimensional and holistic system.^3^^–^^8^ They targeted different levels of DRD severity, subclinical early-stage, mid-stage, and late-stage disease, and identified which variables were potentially ready for current use, or within the next one to two years, and which ones had unmet research needs that could be defined or that were promising but might need a longer than five-year time horizon.

With scientific advancement, a wider variety of targets and mechanisms in DRD can be addressed. New endpoints remain a top priority because our current therapies do not eliminate vision loss. We have non-responders and incomplete responders, and our therapies also have patient and societal burdens in terms of cost, inconvenience and risks. Hence, there is a strong need for new clinical endpoints that address upstream mechanisms and early-stage DRD that can reliably predict clinical benefit. We also need additional clinical primary endpoints to describe the impact of DRD on aspects of vision and patient function that are not currently addressed. The primary goal is to develop new primary endpoints that may be acceptable for use in clinical trials for regulatory approval.

That is a high hurdle, so we have very important secondary goals. Spin-offs of this process may be validation of surrogate or clinical endpoints that can enhance research and clinical care, even if they're not suitable for registration trials. For example, central retinal thickness on optical coherence tomography is not correlated well enough with visual quality of life outcomes to be acceptable as a registration endpoint, yet we use it every day in clinical care and research to look at proof of concept and early efficacy. This validation process will require multiple prospective studies, collaboration across our community with diverse cohorts and defining specific contexts of use.

Robert Levine mentioned assessments of key test probabilities, and the development validation qualification process for these variables entails a better understanding before they get put into long-term and expensive Phase 3 trials. These features include understanding of test variability, their normative data, availability of their floor-to-ceiling effects, subjectivity, and how they change between healthy eyes and eyes with any DRD or late-stage or different stages of DRD so we know what it means to have a real change on these tests. These characteristics are essential to determine whether change over the course of a clinical study is meaningful or relevant. Feasibility concerns, device availability and cost, clinical relevance and patient acceptance and time requirements are also important.

Secondary goals include investigating retinal structure-function relationships, the degree to which measures change with increasing severity of DRD, test-retest variability, correlation, and test characteristics between two eyes of an individual. It is also important to understand whether structural and functional measurements can be validated as surrogate outcomes or clinical endpoints. A treatment study will enroll patients who are starting anti-vascular endothelial growth factor (VEGF) treatment for over four years. A separate treatment study will include 100 patients followed up over one year. The Table shows the ways in which we typically characterize retinal disease in patients in our clinics, eye examination, best corrected visual acuity, a range of imaging including ultrawide fields, color photos, and OCT, a measure of retinal thickness, and OCT angiography, which yields a high-resolution look at the central retinal vasculature. On the right are the procedures we identified as new and promising for this study. We're examining light-adapted and dark-adapted tests so we can identify changes in cone and rod pathways, respectively.

**Table.** Structural and Functional Endpoints for Comprehensive Evaluation of Diabetic Retinal Disease

Standard Endpoints	Functional Endpoints
Eye exam	Manifold quantitative contrast sensitivity (qCSF) (photopic and mesopic)
E-ETDRS visual acuity	Objective field analyzer (M18, W20 tests)
Ultrawide field color fundus photos	RETeval ERG (ISCEV equivalent 3.0 cd s/m^2^ flash, 30-Hz flicker, 63-Hz flicker, Photopic Negative Response protocol)
Ultrawide field fluorescein angiography	RETeval ERG (ISCEV and 3.0 cd s/m^2^ flash equivalent, ISCEV and 0.01 cd s/m^2^ flash equivalent, 1.13 Td flash for OP analysis
SD-OCT	RETeval pupillometry (1-s 300 cd s/m^2^ red and blue flashes)
OCTA	
Humphrey Visual Fields	
Reading speed	
Peripheral blood samples and Urine	
History of systemic co-morbidities	
Social determinants of health	

We highlight tests that rose to the top as we convened expert groups on visual function. The first is quantitative contrast sensitivity testing on the Manifold platform (Adaptive Sensory Technology, San Diego, CA, USA), which applies Bayesian active learning to adjust the contrast and size (spatial frequency) of letter patterns and can be performed across different lighting conditions. The deficits reflect the general dysfunction of rods, cones, and retinal ganglion cells. Data available for patients with diabetes show good repeatability of tests and understanding how these tests change in patients with increasingly severe DRD. The testing time is about four minutes per eye without a chin rest or head rest and a low burden on patients and technicians. The RETeval ERG and pupil response device (LKC Technologies, Gaithersburg, MD, USA) also became a high priority. This device can measure function of bipolar cells and retinal ganglion cells, and the full-field stimulus looks at overall retinal health.

An interesting three-year longitudinal study suggests there is an 11-fold increase in the risk of need for treatment and an ocular intervention due to diabetic retinopathy with abnormalities on this test,^9^ with preliminary data ready in terms of test-retest variability. The testing time here is nine to 10 minutes. Patients will dark-adapt to assess rod photoreceptor function. Third, the Objective Field Analyzer (Konan Medical, Irvine, CA, USA) is an objective visual field test that measures pupillary responses rather than asking patients to indicate when they can see a stimulus. Two protocols, W20 and M18, measure different regions of the retina.

#### Research, Collaboration, and Data Science

Michael Chiang focused on several topics. A major challenge is that our field is subjective and qualitative and a lot of work in AI and ophthalmic imaging addresses these challenges. Several years ago Eric Topol tweeted: “Of all the medical specialties, most people think radiology is leading the AI movement, but it's actually ophthalmology.” One reason for that is OCT imaging, for which David Huang, Jim Fujimoto, and Eric Swanson received the Lasker Award. Technologies like this drive advances in AI, and it's important to recognize that AI, imaging, and clinical data go together.

Using data from these imaging devices, there is a growing literature in the new field of “oculomics” in which ocular imaging biomarkers may have significance (in somewhat preliminary studies) for the diagnosis and prognosis of systemic diseases such as neurodegenerative diseases (e.g., Alzheimer's, Parkinson's), psychiatric disease, cardiac disease, and others.

He addressed the question, “If artificial intelligence is so good, why do doctors still have jobs in 2023?” by highlighting two challenges. One response is that AI systems tend to be very accurate for narrowly defined tasks, yet real-world medical diagnosis is far more complex. For example, the first FDA-approved autonomous AI system in medicine was developed by Michael Abramoff's group at the University of Iowa to identify clinically significant diabetic retinopathy. A second major challenge is generalizability and bias: Clinical research studies are often from relatively homogeneous populations. In the real world, there are multiple imaging devices, different races, different populations. An AI system trained on images from North American babies and tested on images from North American babies works well. But when trained on images from babies from Nepal and tested on images from babies from North America, it worked poorly. However, the AI system works well on a broad dataset of images from North American and Nepalese babies. Thus bias can be a significant problem, and the power of healthcare data can be achieved through large, diverse multicenter data sets.

Large AI-ready datasets are required to avoid the problems of bias. Two major projects at NIH address this bias. Bridge2AI is a trans-NIH project that can involve any field of medicine or science, and has budgeted $130 million over four years to build datasets. One of the four groups that were funded is led by ophthalmologists Aaron and Cecilia Lee at the University of Washington. A second major project is AIM-AHEAD (aim-ahead.net), also a trans-NIH project that budgets $100 million to use machine learning and artificial intelligence to address problems involving health disparities. Goals of this project include building diverse datasets, training a diverse workforce, and developing AI approaches to detect bias. Perhaps the vision field can play a role in this work because ocular images are easy to obtain and are objective, whereas bias often occurs when subjective data are entered into the medical record.

Data sharing and harmonization remain a challenge. A common scenario is that multiple small studies occur in parallel, in which different investigators are addressing the same question, yet the studies are individually underpowered. Different investigators are generally not incentivized to share data in our current academic culture. However, the reality is that even if they want to share, when data are not collected in a harmonized manner, one simply cannot combine datasets.

The NIH data sharing policy is a “stick.” If you don't develop a data sharing plan, we don't fund your research. We need to pair sticks with “carrots” to best incentivize people to share data. The editors-in-chief of leading vision journals meet several times each year to discuss how we might collaborate to advance the community. Out of this effort came a new publication type: a dataset or software library. The idea is to have papers about datasets, with the goal that people would receive academic credit for publishing data as independent standalone products. The first vision journal that implemented this was *TVST* (editor-in-chief Roy Chuck), and the second was *Ophthalmology Science* (editor-in-chief Emily Chew). We need to develop approaches to incentivize data sharing and team science. Furthermore, we lack a common data model for ophthalmology; currently, clinical data in different EHRs are like apples and oranges. At the National Eye Institute (NEI) we are working with the American Academy of Ophthalmology to develop this common data model, led by Sally Baxter, Kerry Goetz, and Michelle Hribar.

There two are broad approaches to sharing data: centralized or federated. The centralized approach brings all data into a common repository (e.g., the American Academy of Ophthalmology's IRIS Registry and the University of Michigan's SOURCE registry). The advantages are that there is simplicity to incorporating everything into a common repository. Disadvantages are the need for extensive data sharing agreements and large data files can be difficult or impractical to transfer. In the federated or distributed data network data stays within the individual sites and the analysis methods are shared (e.g., the European Health Data Evidence Network and PEDSnet, a pediatric network). This approach is more easily scalable because data use agreements are not required. The federated approach requires careful data harmonization.

Interoperability is a significant challenge. Standardized terminologies such as ICD-10, CPT or Systematized Nomenclature of Medicine (SNOMED) whereas EHRs have additional local or proprietary coding systems that make collaboration difficult. Common data models, such as Informatics for Integrating Biology & the Bedside (i2b2), National Patient-Centered Clinical Research Network (PCORnet), and Observational Medical Outcomes Partnership (OMOP) map these coding systems in an overall wrapper and facilitate federated data analysis. The OMOP common data model has gained traction and is used in major projects like All of Us and the European Health Data Evidence Network. This approach allows creation of standardized phenotypes and cohorts, and the use of federated tools to analyze data in a consistent manner. One of my aspirations is for the vision community to develop and adopt these models to support collaboration at scale.

For example, Cindy Cai at Johns Hopkins used OMOP to answer a simple question: the relationship between kidney failure and intravitreal injection of anti-VEGF drugs. In several months, she was able to examine data from 12 sites with 450+ million patients to create a cohort of several million patients with blinding eye disease and answer this question.^10^ This is an example of what can be accomplished through federated analysis and common data models.

Ocular imaging standards are important. Although image data are a basis for the AI revolution in ophthalmology, a major problem is that researchers and clinicians often cannot get access to the raw data because they are locked in proprietary standards. This is an enormous problem for clinical care and research. In radiology, this problem was addressed over 30 years ago by development of the DICOM standard. However, DICOM standards are often not adopted by vendors. This situation is not good for patient care, for research and should not continue. A recent article by Goetz et al.^11^ describes how the FDA and NEI can shift the ophthalmic imaging community toward adoption of standards to facilitate better research and clinical care.

Discussion regarding endpoints included comments by Dolly Chang, Maureen Maguire and Stela Vujosevic that current endpoints for diabetic macular edema (DME) and DR trials, best corrected visual acuity and the Diabetic Retinopathy Severity Score, address late-stage disease processes. However, diabetes is complex, involving neurodegeneration and ischemia so we need additional endpoints that reflect the full disease spectrum and treatments’ impact of various processes. Technological advancements promise more sensitive methods for detecting and monitoring disease progression. Ryan Barunas emphasized that he obviously had progressive DRD before it was diagnosed at late stages. Ramin Tadayoni pointed out that we now do not connect endpoints like contract sensitivity or small scotomas to anything in real life. We must link new endpoints with something real in patients’ lives so they have value in terms of patient impacts, reimbursement, and incentivize investment in research. Sandra Puczynski noted critical aspects of the patient experience to capture aspects like pain and anxiety related to treatments, fear of vision loss and loss of independence, and the idea that treatments for DRD can be very difficult and result in hopeless feelings for patients in a vacuum of dispassionate care. These features speak to how patients feel, and how patients function in ways that we don't currently capture. Thus, there is an unmet need to either develop new patient‐reported outcome measures (PROMs) or modify existing validated ones in so they capture changes in the early stages of the disease, elicit differences between different severity levels of clinical DRD, and demonstrate longitudinal validity in terms of responsiveness to interventions. New PROMs need validation for early structural or functional changes that precede visual acuity changes.

### Ocular Biorepository and Resource Center

#### Ocular Tissue Biorepositories

Patrice Fort discussed the Biorepository & Resource Center (BRC) at the University of Michigan, acknowledging Kelli Ramos, Corey Porter, and Carmen Yu. They have developed standard operating procedures with seven eye banks that cover 21 states, collect medical information, and complement them with in-house phenotyping, including fundus photographs and OCT.

The protocol includes isolating the fovea from the macula, the peripheral retina and the vitreous to correlate clinical phenotyping to multi-omics analysis, including transcriptomics, proteomics and lipidomics to get broad assessment of changes that occur over the course and progression of DRD. The goal is to identify new therapeutic targets through analyses of common changes in region-specific retinal changes through molecular biomarkers identified through multi-tissue and multi-stage correlations and follow up with target validation and target relevance assessments. John Holmes at the University of Pennsylvania who performs the bioinformatics for the Network for Pancreatic Organ Donors with Diabetes (nPOD) and the Cardiovascular Repository for Type 1 Diabetes (CaRe-T1D), provides a similar role for the BRC and will create translatable data between platforms.

Mark Atkinson emphasized the critical role of biobanks to move complications research forward. The nPOD began in 2007 and now works with all 56 procurement organizations in the United States. However, only ∼14,000 of 2.5 million people who die yearly in the United States become solid organ or tissue donors. The nPOD supports about 320 projects in 21 countries.

Now it is known that individuals with type 1 diabetes have 25 to 35% smaller pancreases than age and BMI-matched individuals and within two years of disease onset, they are a third to half the size of normal pancreases. nPOD-supported studies have shown that as little as 50% *β* cell loss can cause type 1 diabetes. Moreover, many patients retain some *β* cell function (C-peptide production) after as much as 50 years after diabetes diagnosis. Thus type 1 diabetes is a heterogeneous disease.

The leadership of NIH, NIDDK, and NHLBI have created a new program, CaRe-T1D. Investigators can choose slides from 60,000 samples and pick what they want. Initially, the predominant way of studying tissues was classical immunohistochemistry, and now it's AI and single cell sequencing.

Chris Gates explained that an essential challenge in biorepositories is having the right samples. Another challenge is that the scope of what one can do with those samples in terms of the analysis is changing. Data repositories build bridges between researchers and data, but these are moving targets, which is extraordinarily difficult. Data repositories generate new types of data that are not organically compatible or computable, which limits the ability to translate them into actionable outcomes.

Fabio Baschiera discussed how drug candidates for DRD are often dismantled relatively late in the development stage. Thus predictive biomarkers, with a human repository to help select the most suitable compound earlier, would be more effective in candidate selection. We need a way to develop drugs that consider the correlation between the biomarkers and the functional effect in patients. A tailored and focused approach to the use of human tissues would improve the success rate of translating results to patients.

Chris German and George King cautioned that biases in repository tissue sources, such as gender and racial background, must be considered. Arup Das emphasized the phenotype of DRD differs widely between American Indians, African Americans, and Caucasians.

Chris Rhodes stated we're at the first wave of maturity of data repositories, and the newer functionality will not just capture information about the sample but also include the molecular or the clinical assay information that was part of the experimental design. Success in cancer has resulted from using the Oncomine database, the cBio platform, and The Cancer Genome Atlas.

#### The MTM Vision Consortium

The Mary Tyler Moore Vision Initiative is an idea sprung, from the hearts of Robert and Mary, that has infected everyone. We are establishing a pre-competitive consortium to enable groups to work together, with the University of Michigan as the organizing legal entity. Partners will contribute their knowledge and their questions and get samples, protein samples, tissue biopsies, tissue data, and clinical trial data and to take it into their own shops.

Matthias Kretzler is a nephrologist and co-lead of the Breakthrough T1D Center of Excellence at the University of Michigan. We take care of all organs impacted by T1D, from pancreas to kidney, nerve, and eye. Genes and environment work together because people with the same glycemic control have different damage of their retinas. This is the era of genome scale profiling, and it can transform data into knowledge. In the kidney field we use research biopsies to give us tissue level information.

Blood and, in the kidney field, urine are obviously valuable biomarkers. We identify factors associated with long-term loss of function and bring that back to individual patient level for patient level pathway assessment. The Kidney Precision Medicine Project (https://www.kpmp.org) is an NIDDK-funded research effort where we took this concept forward for people with diabetic kidney disease. Research biopsies are obtained from study participants then we use centers of excellence around the nation to generate multi-scale data sets in spatial organismal structural resolution of the kidney, and define targeted therapies and the framework of continuous discovery and evolvement.

We built an ontology framework to map different data types onto each other, and then develop different data exploration tools. Many of them were co-developed with the open Human Cell Atlas and the HuBMAP Program (https://commonfund.nih.gov/HuBMAP). Raw data is openly accessible and we have a single click through the data use agreement.

We developed an explorer tool which brings in molecular and cell biologists without bioinformatic expertise and are using the spatial molecular mapping revolution currently built with Nils Gehlenborg from HuBMAP Harvard, and Advanced Atlas Spatial Viewer Tool brings spatial metabolomics, proteomics, and transcriptomic data sets into the Atlas. We have identified over 100 different cellular states in health and disease and mapped these states together in their two and three-dimensional context, to identify how cells are transitioning from healthy to stressed.

It is critical to have data available with long-term outcomes. We can map these deep data sets from the current data back into structured information where there are long-term disease strategies available.

The National Center for Advancing Translational Sciences (NCATS) Rare Disease Network for Glomerular Diseases has delivered 14 years of work identifying the molecular profiles active in each patient, from tissue level information into biomarkers, noninvasively captured from the same patients at the same time, so we can link what happens in the tissue, from endothelial cells to blood and urine.

The pathways active in each patient are identified, and the trials target the pathways which are actively destroying organ function. The patients with specifc pathways are brought to the clinical trials, which are active in parallel. Six trials intersect with our patient pool, and we assign the right patients to the right trials. The Prime CKD Consortium is led by Hiddo Lambers Heerspink in Groningen and Marlon Pragnell from ADA was a lead scientist in some of his former roles involved, where we established a diagnostic biomarker panel to identify the right drug for the right patient.

We told our partners of the bilateral concept, “Come on, let's get together. If you all need carefully annotated human data sets with long-term outcomes linked to it, let's generate a genome scale, and we have them on a common table.” We design and develop next rounds of study that we jointly identify in the steering committee, “These are key missing data we need to have.” Now three years later, we are seeing effective therapeutic trials.

Biosamples from patients responding and not responding to treatments allow us to know if the target is or is not adequately addressed by the therapy, an approach that is becoming standard of care. Over 200 investigators across the United States and Europe participate in the Rare Disease Network for Glomerular Disease. These precedents show the way for DRD research.

Carol Oxenreiter, a lay person, emphatically stated it is imperative that we organize consortia so people can work together and make the field move faster. There are successful precedents for consortia in oncology, such as the Oncomine database (oncomine.org), the cBio platform (cbioportal.org), and The Cancer Genome Atlas (https://www.cancer.gov/ccg/research/genome-sequencing/tcga). Thus there is no reason not to form an active consortium for DRD.

Ulrich Luhmann told how consortia can work in ophthalmology. One of the key prerequisites is a level playing field. It's key to develop trust to address these questions together and respect each other's perceptions and views on questions to be successful. In the MACUSTAR consortium (macustar.eu) Bayer, Novartis, Roche/Genentech, and Zeiss and 9 large public/academic partners work successfully together to develop novel endpoints for intermediate AMD. In principle, short-term follow-up clinical biomarkers are needed to validate and measure outcomes in clinical trials. That is a core initial discussion to align on this to bring things together. The challenge is to keep, from a business perspective, interest high enough for such mid- and long-term investment, because decision cycles are short. Interests shift within pharma companies, so it's important that these models highlight the cost-effectiveness aspect and how we serve a common goal most effectively with respective investments from all ends, academia and pharma, coming together aligned toward a common goal.

Phillip Lai related his observation that collaboration in a consortium often yields greater results than individual efforts. The key question is: How can pharma add value to the consortium, and what can it gain in return to ensure continued engagement? Pharma companies can provide not only resources and the technical expertise needed for various projects but can also provide resources to projects they may not have the capabilities for developing or are not prioritizing internally. These resources can be leveraged within the consortium to address challenges or develop areas that are otherwise under resourced within individual companies. Pharma can also lead in establishing standardized coding resources and a common data framework to facilitate the acquisition, exchange, and submission of data. This applies to both clinical trial patient data and imaging data. For example, protocols for capturing imaging and assessing best corrected visual acuity can vary significantly between different clinical trials and even within the same company. These discrepancies need to be harmonized to create an open database that supports better clinical trial design, allow identification of more precise patient subsets, and ultimately improves the efficiency and quality of clinical trials.

Drew Lewis emphasized consideration of the folks who collect the data—make things as straightforward, easy, repeatable and clean as possible so they can that collect more data more efficiently, cost less money, and require less cleaning. Can we get information cleanly and accurately so we can train AI, and ultimately algorithms and ultimately have a great outcome for patients?

Stephen McLeod pointed out the big challenge with consortia is the huge resources to assemble, but once accomplished the goal is to get the information out to everyone as quickly and as broadly as possible. How can we balance the costs to generate, curate, and distribute data when many users don't have the means to contribute to data generation, management, and curation? How can you distribute the information so it gets to individuals with valid needs even if they can't themselves contribute? We've reached the point that we recognize that despite the challenges this sort of pooled, aggregated cooperation is necessary to move our fields forward.

Shelby Unsworth also discussed the challenges of consortia, such as lengthy execution of a contract between academic and industry partners know that it is often a long and painful process. These relationships, who brings what value, and how to de-risk large investments to achieve our outcomes is to keep that purpose in mind from both sides.

Matthias Kretzler discussed how the Renal Pre-Competitive Consortium^12^ leveraged 21 years of work from three networks, where there was already data in the open space which could be easily ingested, and people had deep knowledge of these data. The critical step is people understanding how to communicate with the different stakeholders around the table. Together we identify how to use data, identify the key gaps to use the data, to make key decisions, to go to boards, and defend their target, and to get the approval to go into the clinic, or to phase three trials.

The individual investment, depending on the size of the consortium, can be very modest, certainly compared to what a phase 2A study costs. Trials can be reduced from 384 to 62 patients with this approach. That recoups the investment of the entire Nephrotic Syndrome Study Network from the NIH and Michigan over 14 years.

Ulrich Luhmann discussed using aqueous humor to explore the possibilities and evaluate the value of that matrix. Is it relevant to monitor some effects from back of the eye? How do we do that? We need to link it up with match sampling, aqueous humor and vitreous humor. This has its challenges, but it's being used in trials currently to answer questions like disease monitoring, trying to link imaging biomarkers up with molecular markers by obtaining really high omics like datasets from very small volumes.

Amitha Domalpally stated the Wisconsin Fundus Photograph Reading Center has the EDIC, DCCT, PPOS, and ACCORD curated data sets that are available. There is increasingly strict regulation, at least in some Europen countries regarding use of patient data, including biologic samples, which makes it really complicated.

Matthias Kretzler explained how the KPMP framework has a data enclave, where the data stays locally, then investigators send algorithms to the data in a secure enclave. The algorithm extracts the relevant information from individual patient-level information. The data is aggregated and exported as aggregate data.

The DRCR Retina Network has demonstrated the ability of clinicians and industry to collaborate in drug trials. It currently does not. In summary, an academic-industry partnership to advance

### Regulatory Pathways in Neurodegenerative Ocular Disease

Wiley Chambers explained that the basis for approval decisions comes from the Food, Drug, and Cosmetic Act. It is very explicit and talks about safety and efficacy being determined by adequate and well-controlled investigations. The bottom line is everything else doesn't count. And as much as any of the nonclinical information leads you to some area, or some assay suggests or some mechanism of action suggests, if it's not demonstrated in adequate and well-controlled trials, it doesn't count for approval. If it works and the people that the product is labeled and defined as working in, that's good enough.

Adequate and well-controlled trials are study designs that permit a valid comparison with comparator arms doing similar schedules where the patients can't tell which group they're in, multiple different things in the trial to minimize bias. On the part of the subjects, observers, analysts, people should not know until the trial was over which group they were in, so that they don't give a false sense of efficacy or don't report aspects of safety.

The method of assessment must be well defined and reliable, and that's where this consistency comes in you see people frequently say, “Well, it's not quite good enough, for what does it need to be validated?” What we're looking for is whether it is well defined and is it reliable?

For diabetic ocular conditions, we separate two different indications. We have treatment of DME and treatment of diabetic retinopathy. You can argue that there's overlap between the two; there certainly is, but we think there are significant differences: products may do one, may do the other, or may do both. For diabetic macular edema, visual acuity must improve by at least 0.3 LogMAR. We don't care if it's in high-contrast, low-contrast, or something in between. That works in both directions, not only can you show improvement, but you can also show prevention of loss. You can do it either as a mean change, or you can do it as the percentage of subjects that show improvement.

We accept other measures of visual function, and we usually describe it as visual function, not as visual acuity. Visual field is equally important. We've defined an amount of change in visual field that's clinically important, that being greater than seven decibels. Picking a predefined area, having that area change by seven decibels—and the area must be a definable area, it's not a single point and, we've arbitrarily said, at least five points. Microperimetry, same thing, you're measuring essentially the same thing.

We may take something that's less than that, but only after we see the results of a clinical trial. This is one side of a coin that's benefit to risk, and if we don't know what the risk is, it's very difficult to make that comparison. Although we've said this is unquestionably efficacy, anything less than that we want to balance with what the risks are.

For diabetic retinopathy the ETDRS scale was developed, and it's not the scale that was important; it was its ability to predict what was going to happen 10 to 15 years later. We will take a change on the EDTRS, either measuring both eyes, and the scale was originally developed to be for both eyes. A three-level change in both eyes or for those products that are administered only to a single eye, a two-level change.

There are other anatomic features that we take as far as endpoints. Preventing loss of photoreceptors, we have two products that are currently approved based on loss of photoreceptors. Preventing retinal detachments is good in any situation, so we take that as an endpoint. We'll take prevention of nerve fiber loss as a legitimate clinical endpoint.

The lack of our current neurological endpoints in diabetes is the fact that we haven't had products that can alter them. If there is a product to change neurological function, we'll notice it in what we do.

Lloyd Paul Aiello discussed the challenge to determine the threshold for a clinically meaningful change as it relates to neural degeneration associated with diabetic retinopathy. A key issue is to identify new measures that impact patient function and how to quantify them accurately and reproducibly enough to guide medical decisions. Vision has other important aspects beyond best-corrected visual acuity, which can impact a patient's life. But can we use that measure to provide useful care?

Ramin Tadayoni presented EviRed, a French government–funded project with around 3000 patients. The idea is to propose a new classification to estimate the risk of patients losing vision. A problem with the ETDRS classification is that the literature says that if you're level 55, you have 50% risk of PDR in a year, but in new studies, it's around 10% or 15%, so it's no longer working.

Jason Ehrlich expressed why updating our current understanding of disease progression rates are essential - be it for the DRSS scale, for functional preservation or just change in vision or contrast sensitivity, any of these parameters - in the modern era with modern diabetic systemic therapies, and the current patient populations and the diversity of those patient populations. That's where the current knowledge is lacking. That is, for a new endpoint how long does it take for a clinically meaningful change to occur with or without treatment? Big pharma company projects have to measure proof of concept in the same period of time as an oncology product, or a blood sugar control product, or a medicine for hyperlipidemia. At small biotech firms, investors will take risks to invest in a new mode of delivering a therapy or a novel, impactful biology, and they try to de-risk it through genetics, or through animal models. Biotechs often can't take risks on new endpoint development.

Chris German told of how as a type 1 diabetic of 35 years, he's had frequent visual fields, OCT scans, and other prescriptive assessments, but they lack predictive potential. They can tell me I need to go on therapy but cannot tell me when I should start intervening or provide real guidance for what I can be doing now to prevent the development or progression of disease down the road. Meaningful endpoints that predict disease onset and progression, and guide intervention, would be extraordinarily valuable. Anyone who is developing assessments and interventions should consider the day-to-day impact and risk added to someone who already has a lot of burden managing their diabetes.

Lauro Ojeda explained that another endpoint could be the ability to perform an activity of daily living. He measures mobility by following up people for days to weeks. He measures how well people move in daily life and see how they slow down with medical conditions like Parkinson's.

Arup Das related that post-hoc analysis of the DCCT data showed that hemoglobin A1c explains only 11% of diabetic retinopathy risk reduction.^13^ The DCCT also showed there is familial clustering in development of severe NPDR and PDR. The Wisconsin Epidemiologic Study of Diabetes showed only 50% of type 1 diabetics with long-duration diabetes develop PDR, and the other 50% do not develop in their lifetime. Why?

American Indians rarely develop center-involving DME and rarely need anti-VEGF injections, although they develop PDR, whereas Hispanics develop the worst kind of center-involving DME. This raises the question: are PDR and DME two different disease processes? We found in our DRGen Study^14^ that rare genetic variants “modify” the disease progression in DR. Possibly there are genetic variants that “protect” certain patients from developing severe form of DR. Hopefully, in the near future, genomics data will predict who is going to get DR, who not, and if so, what type of severity, mild, moderate or advanced disease like DME or PDR. Also, we will be able to predict who is going to respond to anti-VEGF injections or not.

Risa Wolf and Julie Rosenthal discussed the emerging understanding that children with type 1 and type 2 diabetes bear the burden of neurodegenerative changes and probably visual function. But we're not really doing anything about it until the kids start to develop retinal hemorrhages. We need earlier endpoints so we can intervene to prevent vision-threatening DRD in their mid-twenties, which is completely unacceptable. About 77 adolescents performed frequency doubling perimetry visual field tests and about 30% of them had abnormalities on that testing but none had clinically evident change when examined. Something is happening before we see any changes.

### Hot Topics

Risa Wolf cautioned that the incidence of type 1 diabetes has increased about 20% over the last two decades, Type 2 diabetes in youth has increased 30%, and these numbers are projected to grow. The TODAY and SEARCH studies^15^^,^^16^ showed a prevalence of 3% to 6% for type 1 diabetes and 9% to 14% in early type 2 diabetes. T1D Exchange data show less than 20% of youth meet the ADA goals for HbA1c of < 7%, so less than 20% are meeting that goal, and they have higher blood pressure, as well as elevated cholesterol. Youth-onset type 2 diabetes has a higher age-adjusted prevalence of kidney disease, and retinopathy, with 9% with DR versus 5% in type 1 diabetes. More than 50% of persons with youth-onset type 2 diabetes have some diabetic retinopathy after about 10 years duration and at a mean age of about 25 years. Eighty percent of the now-young-adults have at least one complication from type 2 diabetes. On a positive note, youth with T1D who use pumps have lower odds of developing diabetic retinopathy.

Compliance with recommended diabetic retinopathy screening is low, particularly in minority and underserved youth. Autonomous AI in the pediatric setting improved the screening rates from 49% to 95%.^17^ Patients were randomized to usual care, which is, “Please go do your eye doctor, get a diabetic eye exam,” or to the AI group, where at the point of care, an AI diabetic eye exam is done, and you get a result in 10 minutes. In the usual care group, 22% of patients completed their diabetic eye exam, and 100% of the kids had it done in the AI group at the point of care.

Paulo Silva described the role of telemedicine for DRD, which relies on remotely acquired retinal images, centralized evaluation of images, and findings communicated to remote sites. Interpretation of these images can be done by AI. Current diabetes and eye treatments combined with telemedicine may reduce the incidence of diabetes blindness by as much as 95%. The population is typically younger, often underserved, and with shorter diabetes duration and less-severe retinopathy. These features contribute to timely ocular and medical management, and we increase the number of patients identified by nearly threefold. Thus reducing barriers to eye care can substantially increase patient surveillance, which likely improves long-term care. Integrated ultra-wide-field imaging and OCT imaging increases the detection of center-involved macular edema by over sevenfold and reduces false-negative rates by 66%.

He argued for a shift to its population-based evaluations, deployment in wide geographic areas and access to diverse patient populations, leveraging less expensive and more convenient imaging devices, allowing broader accessibility of imaging, improved portability and increased patient participation. Systematic evaluation of all diabetes patients increases cost-effectiveness. Over the next two decades, there's a need to address nearly 1.3 billion retinal images yearly so we need to integrate AI at the point of care. by integrating AI at the point of care in a community-based diabetic retinopathy screening program. The goal is to predict development or progression using color photographs with or without systemic risk factors.

He developed and validated a machine learning algorithm for DR progression from 9970 ultra-wide-field retinal images and achieved a classification test AUC of 0.967. These findings demonstrate the accuracy and feasibility of using machine learning models for identifying DR progression developed using ultra-wide-field images. Automated retinal image analysis and deep machine learning will potentially change the way images are evaluated.

### MTM Vision Future View

The effort against DRD includes talent and commitment from beyond the medical community. Nicole Sexton is President and CEO of the Entertainment Industry Foundation (EIF) and Brian Gott is Chief Innovation Officer and Head of Industry Relations. Brian spent 12 years at *Variety*, the leading publication inside the entertainment business as its publisher. Monica Oxenreiter is VP of Content at *Close Concern*s, a blog and newsletter dedicated to the diabetes community. Monica was diagnosed with type one diabetes in 1995. Robert Levine was the executive producer of a beautiful award-winning documentary, *Being Mary Tyler Moore*, which won the Critics Award Association for Best Documentary. Mary had a huge, long-lasting effect with the most prolific studio in the history of television. The opportunity here is to create an understanding of finding cures and interventions around the Mary Tyler Moore's Vision Initiatives' goals and mission and to increase understanding, empathy and awareness of diabetes.

EIF is the 32nd largest nonprofit in the country that no one's ever heard of. EIF runs different campaigns like Idol Gives Back, Stand Up To Cancer, Hope for Haiti, One Hand, One Heart, Defy: Disaster, and Delivering Jobs. It was started in 1942 by Sam Goldwyn and the Warner Brothers.

The philanthropic initiatives of people like Charlize Theron, George Clooney, Colin Kaepernick, Cher, and others come to EIF to develop their strategies for giving back and rely on us to provide fiscal sponsor services for their efforts. In 2022, we placed $250 million in donated advertising assets across network, syndicated and streaming television, national and local market radio, and out-of-home. We work with the Mary Tyler Moore Vision Initiative to create compelling campaigns to drive new audiences, create awareness, new donation vehicles, and fund research that will find that cure for vision loss as it relates to diabetes.

Monica Oxenreiter works at *Close Concerns,* a news organization that covers everything about diabetes and obesity in our newsletter that goes out to 10,000 healthcare teams. *Closer Look*, the newsletter, has the goal so readers can know everything that's happening in the landscape.

### Summation and Next Steps

Lloyd Paul Aiello noted that since 2022 the growth and progress of the MTM Vision Initiative has been remarkable, yet, there is still so very much to do. The composition of the audience is an amazing diversity of interests, and of all incredibly high caliber. The list of attendees in the program comprises 57 pages of notable accomplishments, and some good headshots as well! Martin started the meeting by saying that he's looking to build super groups with the concept that the whole is greater than sum of the parts—a phrase you have heard several times today. Given the “parts,” the greater sum of the parts is going to be something truly remarkable indeed.

Robert described how we need to accelerate development of new therapies to preserve and restore vision and how he hopes that this organization can be a catalyst to that effort. Robert showed a side of how phones evolving from rotary dial phones to the latest computers we have in our pockets. When this initiative started, it was more like communication by smoke signals, progressing to Morse code, and then the rotary phone. It is breathtaking to consider what may lie in store if we put our minds to the task and keep moving along in this manner.

Amazing progress setting up working groups, defining study approaches, delineating requirements and a natural history clinical study poised to start. The study will provide us with remarkable new information on visual function metrics in diabetic and nondiabetic individuals.

Mike Chiang talked about the importance of large data and how the eye can be a window to the body. Thus what we do here will have ramifications far outside of DRD. What remarkable progress in a short period of time having established, equipped, and trained people, and developed multiple SOPs in nine eye banks in over 23 states. Mark Atkinson summarized how important this type of repository of human tissues will be as we focus on disease complications.

The regulatory session discussion had lots of thought and questions about how we might eventually develop new areas to evaluate and how these might be used. Dr. Chambers defined what criteria are now accepted and the type of things we need to think about if we're going to find other acceptable endpoints. Now it's our collective need to carefully and creatively think about how we move forward, especially as the initial clinical trials begin. These efforts will be critical to eventually define accepted new endpoints of visual function.

The Hot Topics presentation described the prevalence of diabetes and diabetic retinopathy in other than the adult population, particularly regarding type 2 diabetes in children, a fact hardly recognized in prior decades.

Additional discussions covered the efforts involving PROMs and how they are needed and what remains to be done. This area will become ever more important to future clinical trials which will need to include them. Telemedicine and particularly artificial intelligence were covered. Undoubtedly, AI is rapidly becoming more important and highly diversified and yet requires great care during development. AI will radically change how we do many components of research and clinical care, particularly considering the 1.3 billion images per year required to assess diabetic retinopathy alone.

In summary, an amazing mind trust has been dedicated to this initiative, and amazing accomplishments have already occurred in a short time. The start of this initiative reminds me of over 20 years ago when we started the DRCR. DRCR started with a telephone call between five people in different locations—Rick Ferris from the NEI, Don Everett the NEI program coordinator, Matthew Davis, at the Reading Center at the University of Wisconsin, Roy Beck at the Coordinating Center in Tampa, and me at Joslin as DRCR chair. We had a concept: to make a difference and to do things no one had done before. After that call we agreed to one thing, namely that any group participating in the consortium must have access to this newfangled technology called the internet. This decision allowed us to start with electronic case report forms and add assisted decision-making to the clinical trial experience. Very new at the time, but it enabled us to quickly do much more complex studies that would have been impossible otherwise. Then the DRCR took off, and the Mary Tyler Moore Vision Initiative is at that stage where it's starting to take off.

In conclusion, a special thanks to Robert and to Mary Tyler Moore for their vision and inspiration and guidance, culminating in this most important initiative. Like Mary Tyler Moore herself, who affected so many people in so many ways, this program has great potential to exert a tremendous impact on diabetes care around the world, and in so doing make the world a kinder and more beautiful place for all with diabetes and those who love them.

## References

[1] Sun JK, Aiello LP, Abramoff MD, et al. Updating the staging system for diabetic retinal disease. *Ophthalmology*. 2021; 128: 490–493. 33218709 10.1016/j.ophtha.2020.10.008 PMC8378594

[2] Jampol LM, Tadayoni R, Ip M. Need for a new classification of diabetic retinopathy. *Retina*. 2021; 41: 459–460. 33315822 10.1097/IAE.0000000000003070 PMC7889282

[3] Glassman AR, Elmasry MA, Baskin DE, et al. Visual function measurements in eyes with diabetic retinopathy: an expert opinion on available measures. *Ophthalmol Sci*. 2024; 4(5): 100519. 38881606 10.1016/j.xops.2024.100519 PMC11179417

[4] Hartnett ME, Fickweiler W, Adamis AP, et al. Rationale of basic and cellular mechanisms considered in updating the staging system for diabetic retinal disease. *Ophthalmol Sci*. 2024; 4: 100521. 39006804 10.1016/j.xops.2024.100521 PMC11245984

[5] Mellor J, Jeyam A, Beulens JW, et al. Role of systemic factors in improving the prognosis of diabetic retinal disease and predicting response to diabetic retinopathy treatment. *Ophthalmol Sci*. 2024; 4(4): 100494. 38694495 10.1016/j.xops.2024.100494 PMC11061755

[6] Tan TE, Jampol LM, Ferris FL, et al. Imaging modalities for assessing the vascular component of diabetic retinal disease: review and consensus for an updated staging system. *Ophthalmol Sci*. 2024; 4: 100449. 38313399 10.1016/j.xops.2023.100449 PMC10837643

[7] Channa R, Wolf RM, Simo R, et al. A new approach to staging diabetic eye disease: staging of diabetic retinal neurodegeneration and diabetic macular edema. *Ophthalmol Sci*. 2024; 4: 100420. 38284099 10.1016/j.xops.2023.100420 PMC10818256

[8] Vujosevic S, Chew E, Labriola L, Sivaprasad S, Lamoureux E. Measuring quality of life in diabetic retinal disease: a narrative review of available patient-reported outcome measures. *Ophthalmol Sci*. 2024; 4: 100378. 37868790 10.1016/j.xops.2023.100378 PMC10585645

[9] Brigell MG, Chiang B, Maa AY, Davis CQ. Enhancing risk assessment in patients with diabetic retinopathy by combining measures of retinal function and structure. *Transl Vis Sci Technol*. 2020; 9: 40. 10.1167/tvst.9.9.40 PMC7453041 32908803

[10] Cai CX, Nishimura A, Bowring MG, et al. Similar risk of kidney failure among patients with blinding diseases who receive ranibizumab, aflibercept, and bevacizumab: an observational health data sciences and informatics network study. *Ophthalmol Retina*. 2024; 8: 733–743. 38519026 10.1016/j.oret.2024.03.014 PMC11298306

[11] Goetz KE, Reed AA, Chiang MF, et al. Accelerating care: a roadmap to interoperable ophthalmic imaging standards in the United States. *Ophthalmology*. 2024; 131: 12–15. 37978977 10.1016/j.ophtha.2023.10.001

[12] Tomilo M, Ascani H, Mirel B, et al. Renal Pre-Competitive Consortium (RPC(2)): discovering therapeutic targets together. *Drug Discov Today*. 2018; 23: 1695–1699. 29778696 10.1016/j.drudis.2018.05.021

[13] Lachin JM, Genuth S, Nathan DM, Zinman B, Rutledge BN. Effect of glycemic exposure on the risk of microvascular complications in the diabetes control and complications trial—revisited. *Diabetes*. 2008; 57: 995–1001. 18223010 10.2337/db07-1618

[14] Cabrera AP, Monickaraj F, Rangasamy S, Hobbs S, McGuire P, Das A. Do genomic factors play a role in diabetic retinopathy? *J Clin Med*. 2020; 9(1): 216. 31947513 10.3390/jcm9010216 PMC7019561

[15] Bjornstad P, Drews K, Zeitler PS. Long-term complications in youth-onset Type 2 diabetes. Reply. *N Engl J Med*. 2021; 385: 2016. 34788520 10.1056/NEJMc2114053 PMC8957477

[16] Jensen ET, Rigdon J, Rezaei KA, et al. Prevalence, progression, and modifiable risk factors for diabetic retinopathy in youth and young adults with youth-onset Type 1 and Type 2 diabetes: the SEARCH for Diabetes in Youth Study. *Diabetes Care*. 2023; 46: 1252–1260. 37043887 10.2337/dc22-2503 PMC10234751

[17] Wolf RM, Channa R, Liu TYA, et al. Autonomous artificial intelligence increases screening and follow-up for diabetic retinopathy in youth: the ACCESS randomized control trial. *Nat Commun*. 2024; 15: 421. 38212308 10.1038/s41467-023-44676-z PMC10784572

